# Supplementary material for: Revealing the hidden third dimension of point defects in two-dimensional MXenes
Source: Nat Commun. 2026 Apr 14;17:3473. doi: 10.1038/s41467-026-71670-y (PMC13079715; doi:10.1038/s41467-026-71670-y)
Supplement: Supplementary file 1 — Supplementary Information [file 41467_2026_71670_MOESM1_ESM.pdf]

Supplementary Information

Revealing the Hidden Third Dimension of Point Defects in Two-Dimensional MXenes

Grace Guinan, Michelle A. Smeaton, Brian C. Wyatt, Steven Goldy, Hilary Egan, Andrew Glaws, Garritt J. Tucker, Babak Anasori, and Steven R. Spurgeon

Contents

Supplementary Note 1: Configuration and Validation of ML Models

Supplementary Figures

- Supplementary Figure 1: ML Pipeline
- Supplementary Figure 2: Model Performance vs. Number of Aligned Images
- Supplementary Figure 3: Layer Deconvolution
- Supplementary Figure 4: Delaunay Triangulation
- Supplementary Figure 5: Bootstrapping Analysis
- Supplementary Figure 6: Vacancy Percentage Comparison
- Supplementary Figure 7: XRD Measurements
- Supplementary Figures 8-9: Additional Modeling Figures
- Supplementary Figures 10-12: MXene STEM Images

Supplementary Note 1: Configuration and Validation of ML Models

Here, we provide a visual of our model training and validation process. Absent ground truth data, it is challenging to validate model performance. It is possible to create simulated data; however, making simulated data resemble experimental data is imprecise and can introduce significant bias. We chose to conduct expert comparison of the performance of our model to 2D Gaussian Fitting (the method we used to train the model). As shown in Supplementary Figure 1b, 2D Gaussian Fitting does not provide informative or regular atomic positions (and cannot find defects). In comparison, our NN enforces a regular, hexagonal lattice, can capture the locations of defects (even for a large cluster of defects, as in the 12.5% HF sample), and is more robust to noise in low-dose images.

Supplementary Figure 1: ML Pipeline

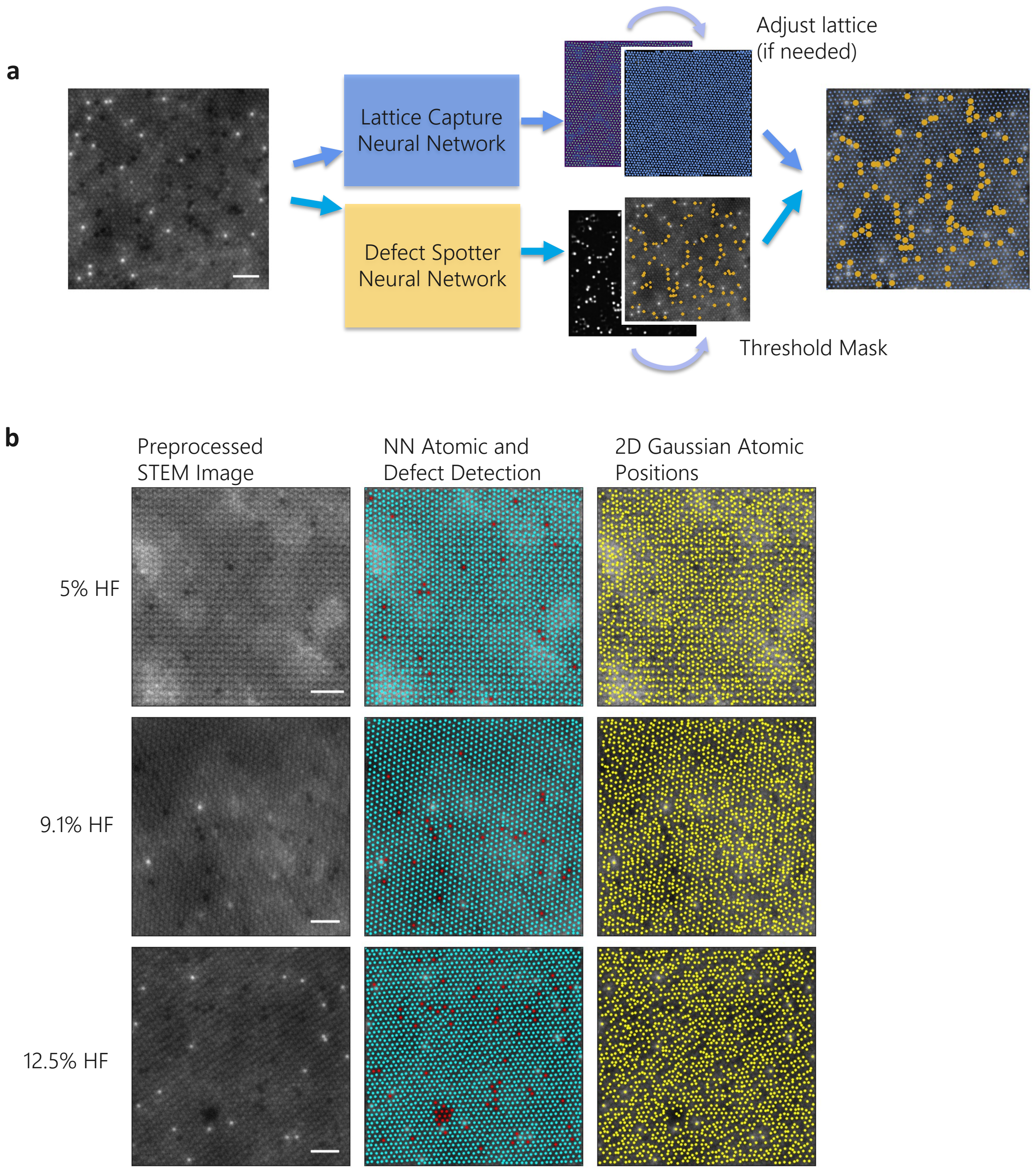

Supplementary Figure 1. **Model training and performance. a** Model pipeline. STEM-HAADF images are run through two different neural networks, one to find all atomic positions by enforcing a hexagonal lattice structure (Lattice Capture) and another specifically to detect defect positions (Defect Spotter). Models are combined to find final atomic and defect positions. **b** Performance of our model for three sample images in comparison to 2D Gaussian Fitting. Atomic (blue) and defect (red) positions are visually more regular in comparison to atomic positions found using Atomap's[1] 2D Gaussian Fitting Model (yellow). Scale bars 1 nm.

Supplementary Figure 2: Model Performance vs. Number of Aligned Images

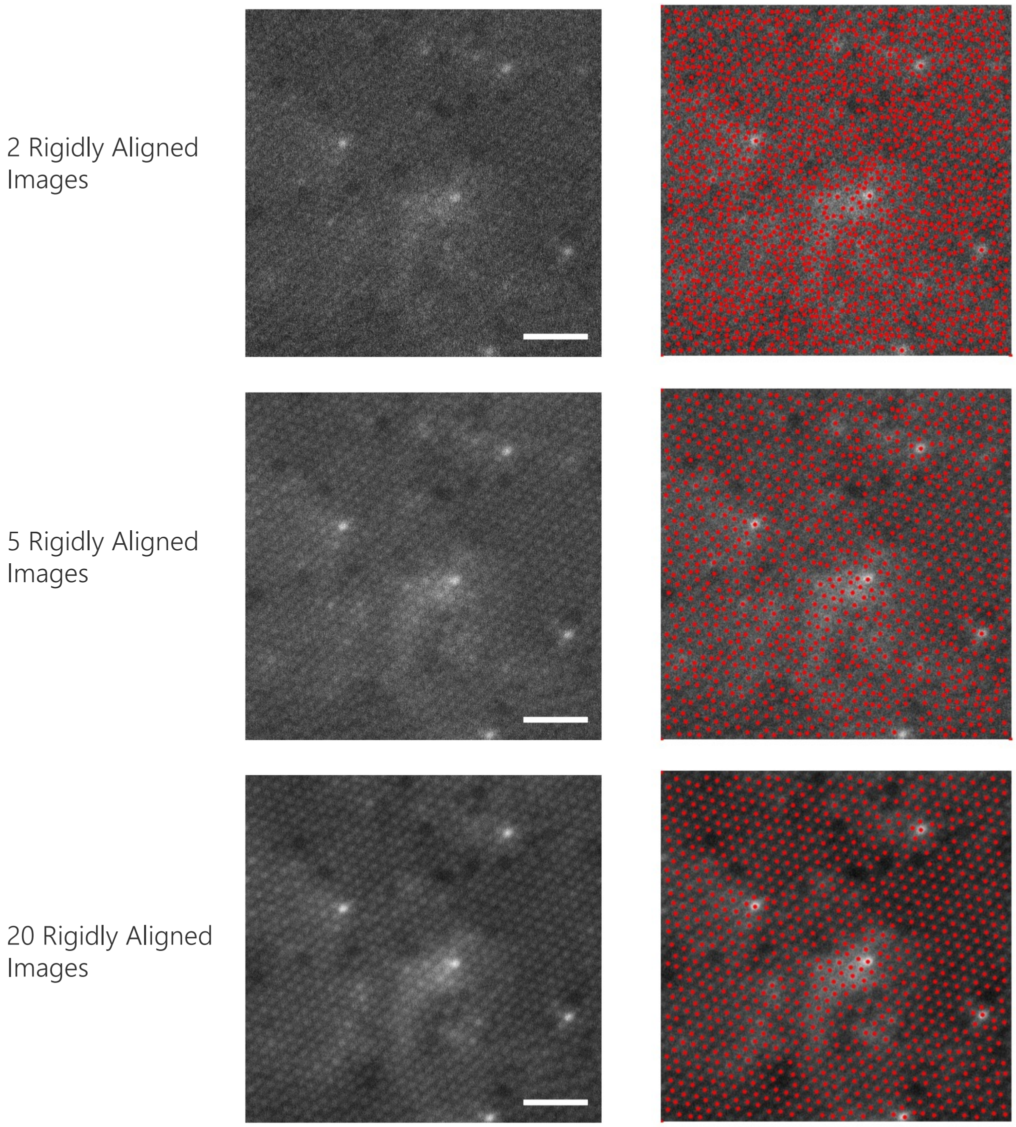

Supplementary Figure 2**. Rigid Alignment of Images.** To achieve higher effective resolution, raw images were rigidly aligned and summed as described in Methods. Model performance is shown as a function of the number of aligned images. Red dots indicate atomic positions found by the model. While slight atomic movements are observed across the raw frames, image stacking was necessary to achieve the performance shown in the bottom-right panel. Future work will explore models capable of operating directly on extremely low–signal-to-noise images, focusing on time-resolved analysis rather than rigidly aligned averaging and allowing us to investigate defect dynamics. Scale bars 1 nm.

Supplementary Figure 3: Layer Deconvolution

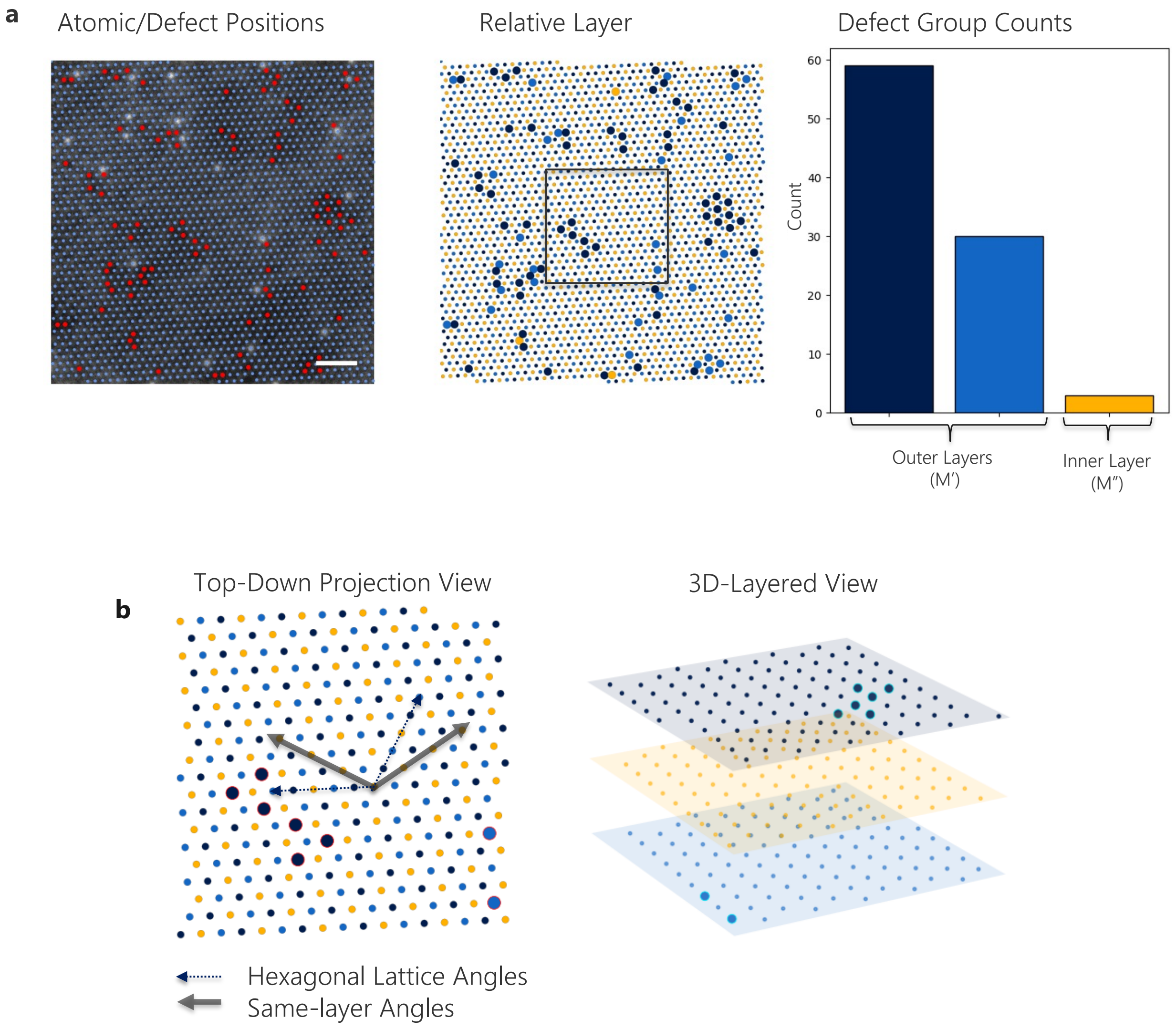

Supplementary Figure 3. **Layer deconvolution method. a** Steps to identify outer (M') vs. inner (M") layers, given atomic (blue) and defect (red) positions. Dots are grouped by relative layer and histogram of defect groups reveals outer vs. inner layers. Scale bar 1 nm. **b** Close up of relative layer labeling. When looking at $Ti_3C_2T_x$ MXene in top-down projection view, colored by layer, same-layer angles are 30 degrees off from hexagonal lattice angles. These three layers can be discriminated in the 3D-Layered View with outer layers M' (light/dark blue) and middle layer M" (orange).

Supplementary Figure 4: Delaunay Triangulation

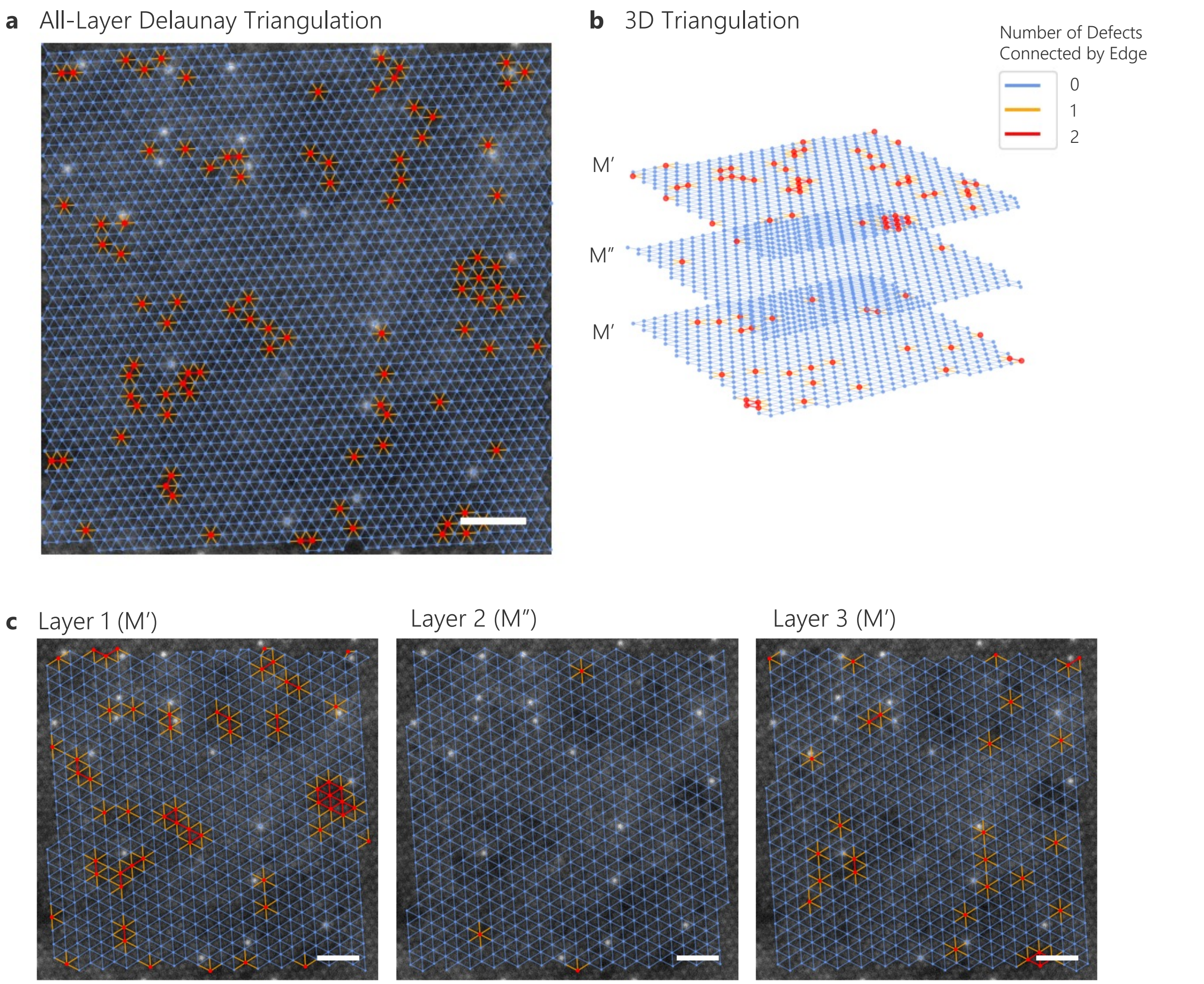

Supplementary Figure 4. **Delaunay triangulation to identify motifs. a** Delaunay triangulation on all atomic and defect positions. Red lines indicate adjacent defects, yellow lines indicate edges that are connected to one defect, and blue lines indicate edges between atoms. This is across all the three layers. **b** Visualizing Delaunay triangulation in 3D, showing how we can now compare defects within layers. **c** Separating out the three layers. All colors indicate the same edge conditions, however, now we measure this within layers (e.g. adjacent defects in the top layer). By applying the Delaunay triangulation, we can understand how defects form within and between layers. Scale bars 1 nm.

Supplementary Figure 5: Bootstrapping Analysis

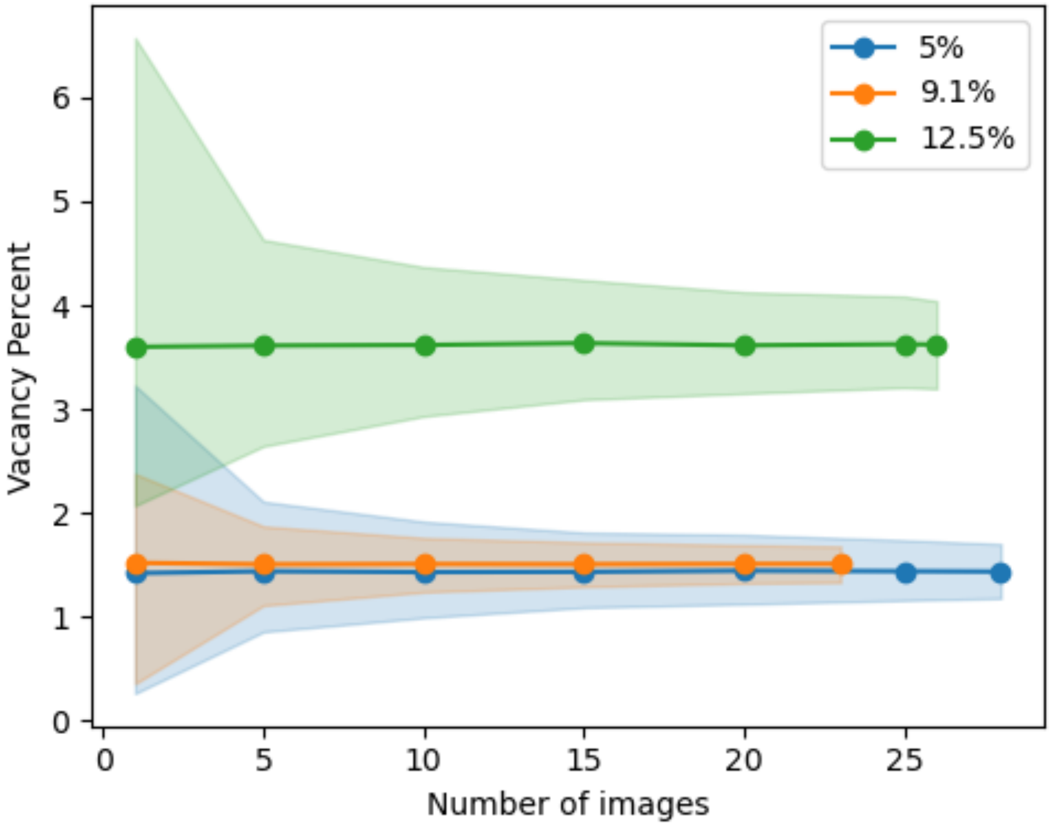

Supplementary Figure 5. **Bootstrapping across number of images.** Bootstrapped vacancy percentage calculations for images sampled from each HF concentration: 5% (blue), 9.1% (orange), 12.5% (green). Filled dots show the conditions where bootstrapping analysis was conducted (B = 2000) and shaded bands indicate a 95% confidence band, calculated by taking the 2.5 and 97.5 percentiles of the bootstrapped distributions. The 95% confidence interval narrows as image count increases for all three samples, differentiating the vacancy concentration in the 12.5% HF sample from 5% and 9.1% HF samples. However, the intervals of the 5% and 9.1% HF samples are still overlapping.

Supplementary Figure 6: Vacancy Percentage Comparison

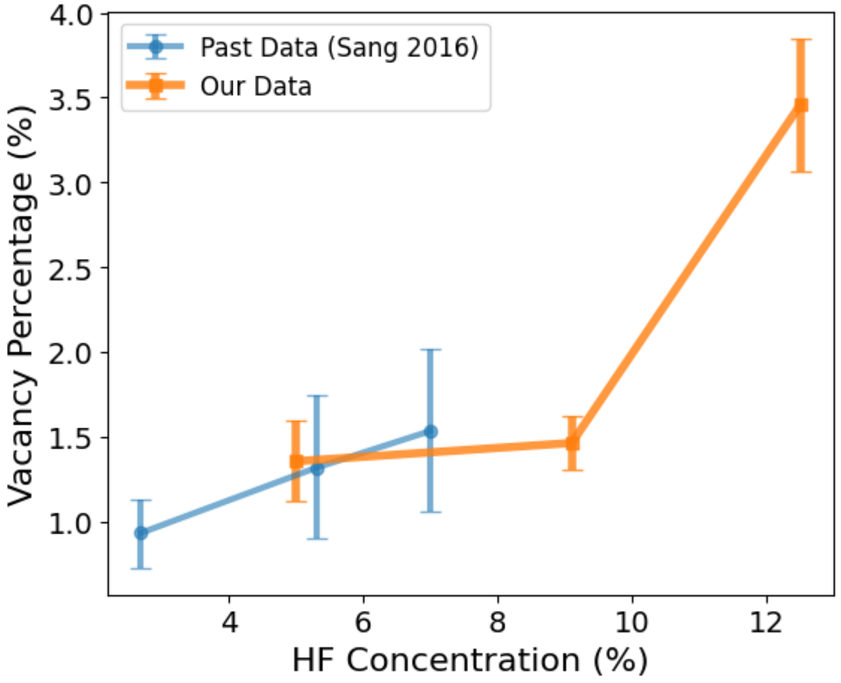

Supplementary Figure 6. **Comparison of Vacancy Percentage to Past Study.** The vacancy percentages found in this study (orange) are compared to a previous study[2] (blue), which confirms the trend seen in our study. Mean values and standard deviations from previous study are digitized using Plot Digitizer (version 3.1.6)[3]. Filled-in dots represent the mean vacancy concentration for each HF concentration: 2.7, 5.3, 7% (previous study) and 5, 9.1, 12.5% (our study). Orange error bars represent the 95% confidence intervals calculated in S5 (bootstrapping for all the images in each dataset).

Supplementary Figure 7: XRD Measurements

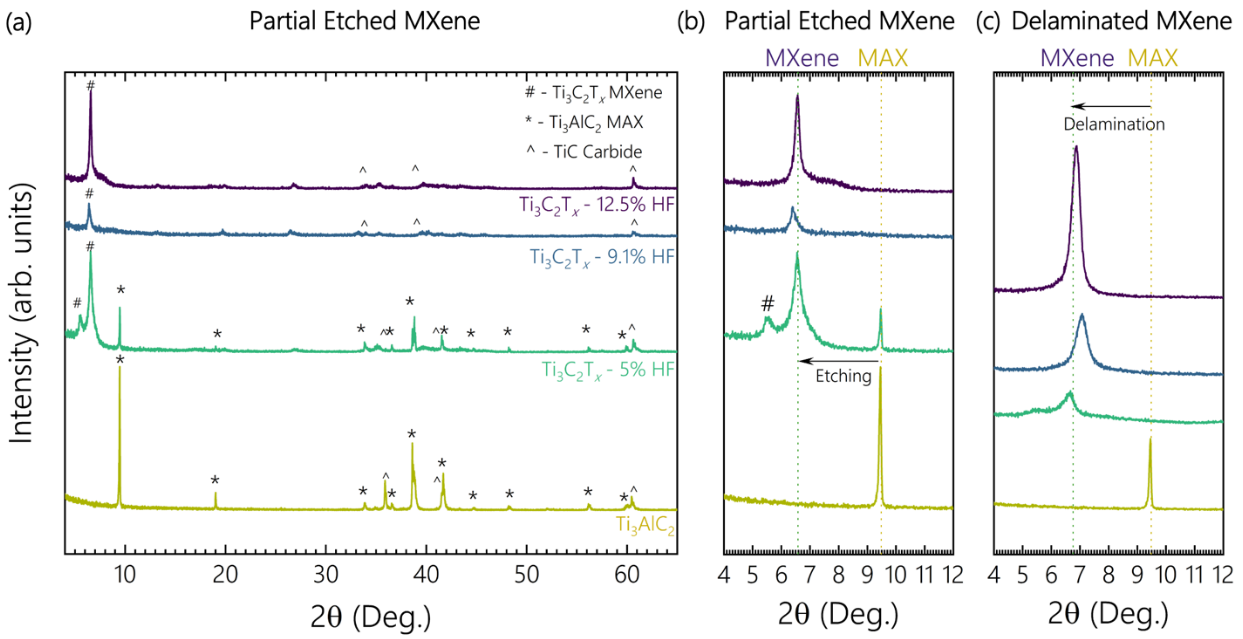

Supplementary Figure 7. **X-ray diffraction (XRD) of $Ti_3C_2T_x$ MXenes from the Ti_3AlC_2 used in this study. a,b** The $Ti_3C_2T_x$ MXenes synthesized using 5% HF demonstrate partial etching, as shown by the presence of both MAX and MXene peaks, while the 9.1 and 12.5% HF show only MXene after etching. As the MXenes undergo a delamination process, the **c** final fully etched $Ti_3C_2T_x$ MXenes used in this study for imaging were separated from both remaining MAX and impurity phases.

Supplementary Figures 8-9: Additional Modeling Figures

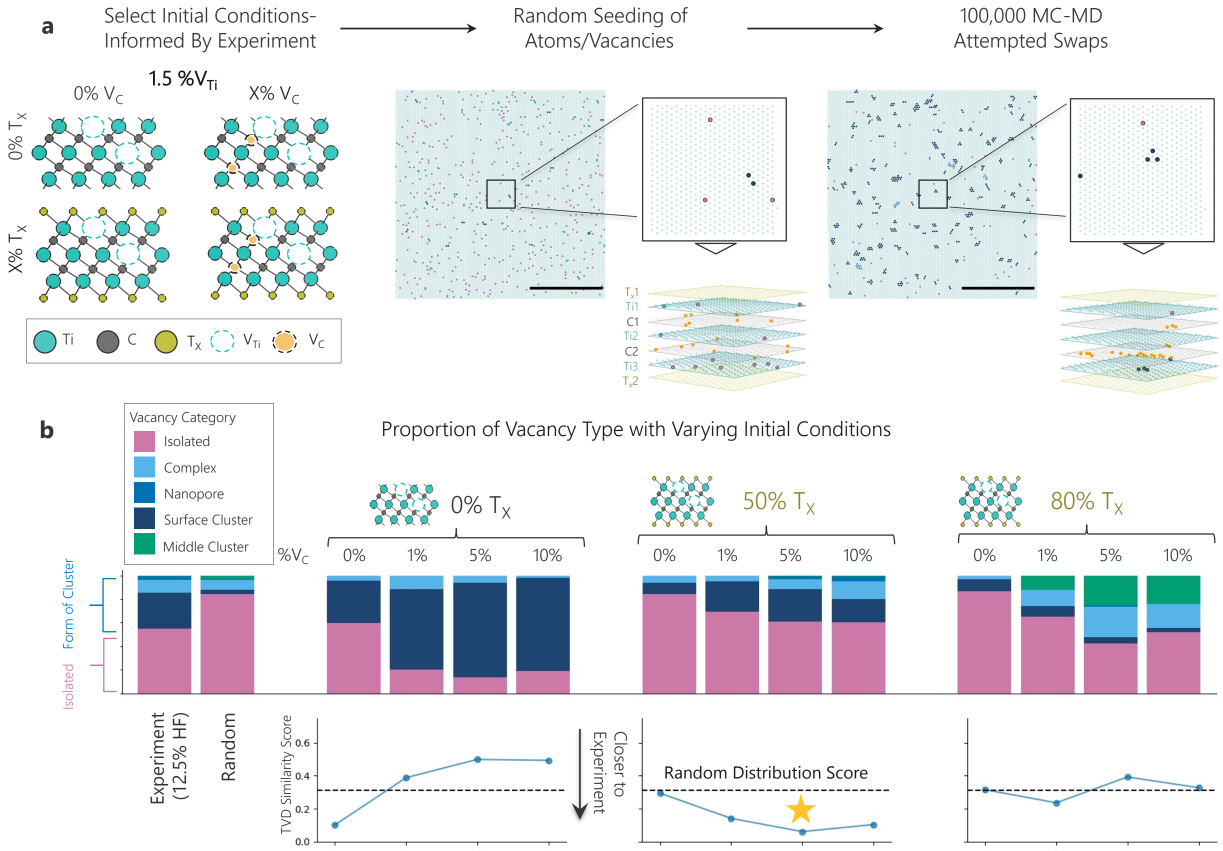

Supplementary Figure 8. **1.5 V_{Ti} MCMD results.** **a** Workflow for Monte Carlo (MC)–Molecular Dynamics (MD) simulations. A V_{Ti} concentration of 1.5% (from 5% and 9% HF experiments) was used. Candidate $Ti_3C_2T_x$ grids were seeded with varying V_C concentrations (0, 1, 5, 10%) and surface termination (T_x) levels (0, 50, 80%). Example shown in random and final MC step configurations (scale bars: 10 nm). Bolded dots represent vacancies, color indicates vacancy category; C vacancies (orange) and Ti vacancy categories, including isolated (pink), complex (light blue), nanopore (medium blue), surface cluster (dark blue), and middle cluster (green). **b** Vacancy clustering in relaxed configurations compared with experiment and random. Line plots show the Total Variation Distance (TVD) between clustering distributions from the 12.5% HF sample and corresponding MC–MD simulations shown directly above each point, with the random TVD score (dotted line) as reference. Y axis is shared across the line plots. Yellow star indicates run with the best TVD score (50% T_x, 5% V_C).

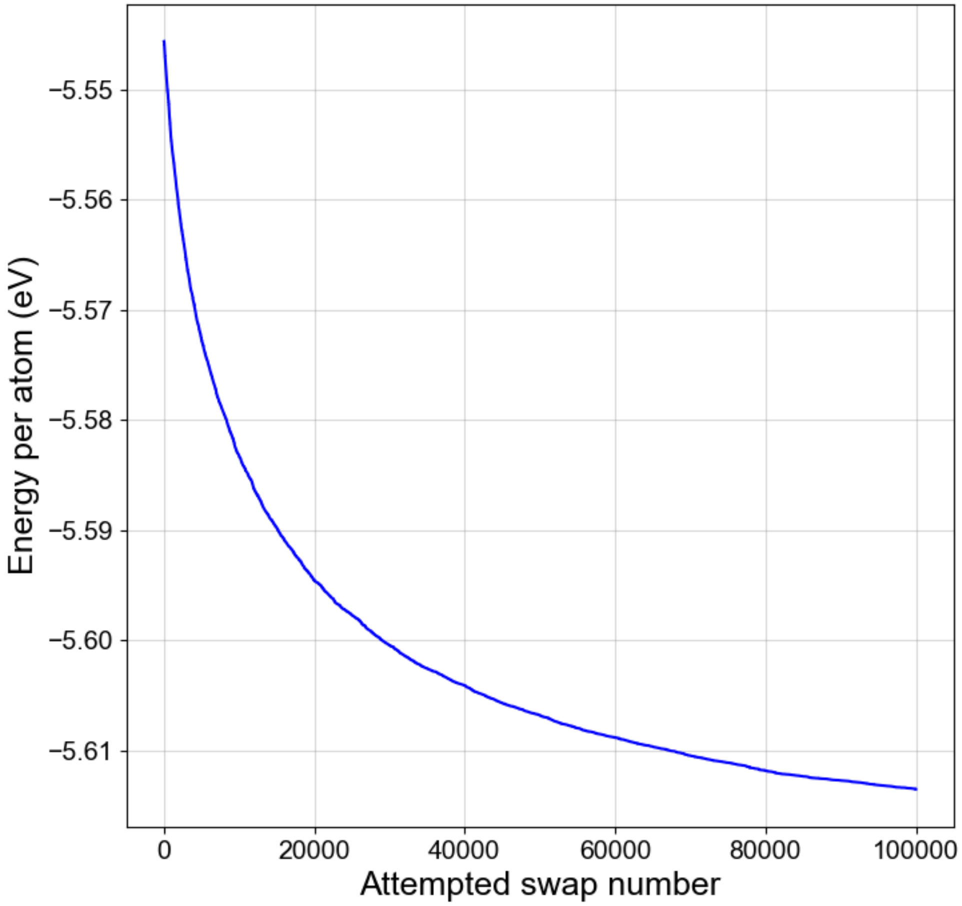

Supplementary Figure 9. **Energy curve across MC-MD swaps.** The MC-MD algorithm attempts to lower the overall energy in the system through random swaps of atoms and "ghost" vacancies. Here, we choose to run the MC-MD until the overall energy in the system flattens out, around 100,000 attempted swaps.

Supplementary Figures 10-12: MXene STEM Images

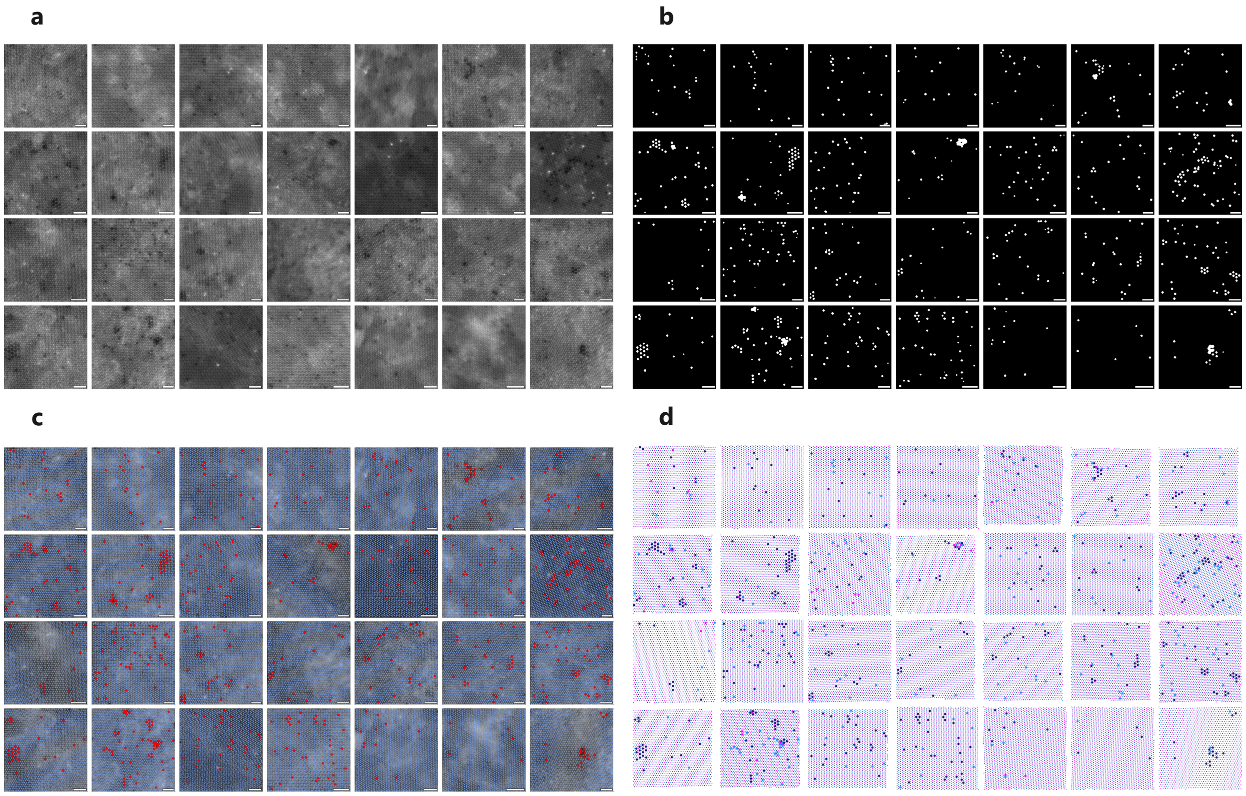

Supplementary Figure 10. **5% HF MXene images. a** STEM images of MXenes etched with 5% HF. **b** Neural network-outputted masks from the defect finder NN. White blobs indicate predicted defects. **c** All located atoms (blue) and defects (red). **d** Atoms and defects colored by layer; M'_{max} (dark blue), M'_{min} (light blue), M" (pink). Scale bars 1 nm.

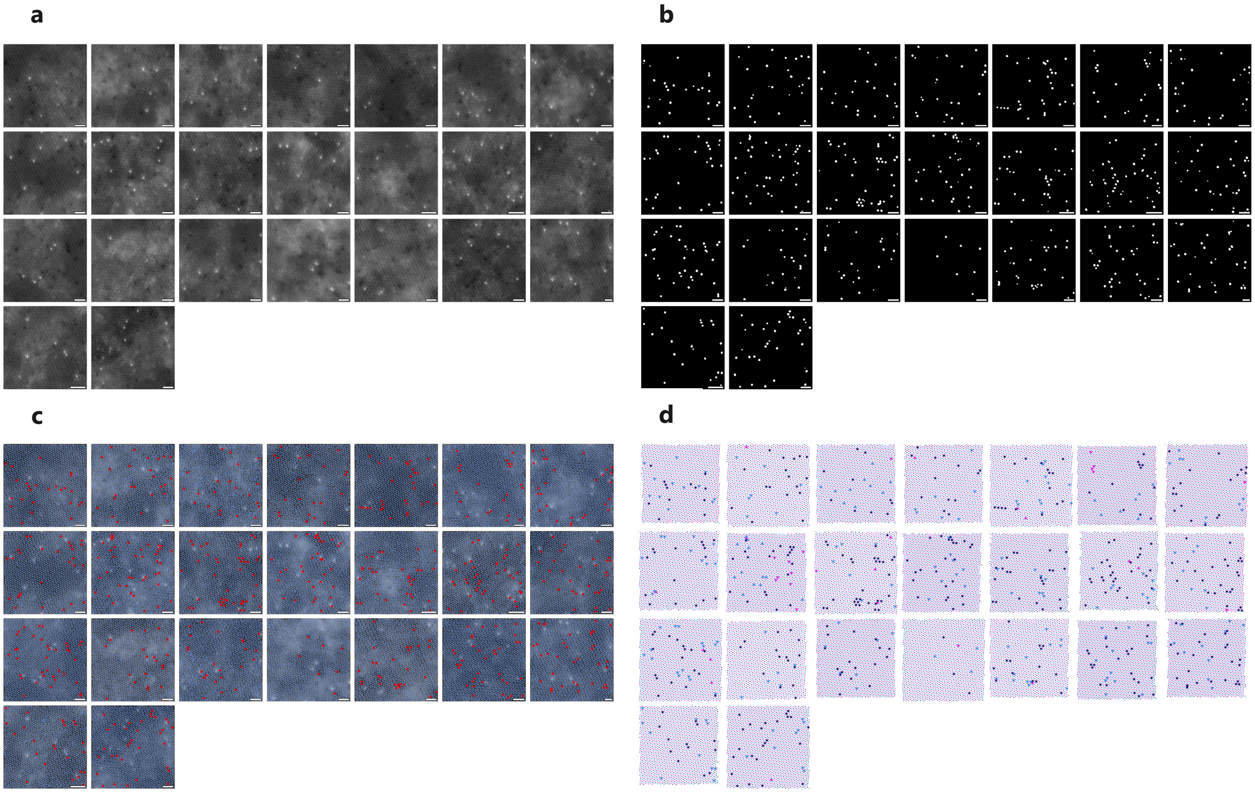

Supplementary Figure 11. **9.1% HF MXene images. a** STEM images of MXenes etched with 9.1% HF. **b** Neural network-outputted masks from the defect finder NN. White blobs indicate predicted defects. **c** All located atoms (blue) and defects (red). **d** Atoms and defects colored by layer; M'_{max} (dark blue), M'_{min} (light blue), M" (pink). Scale bars 1 nm.

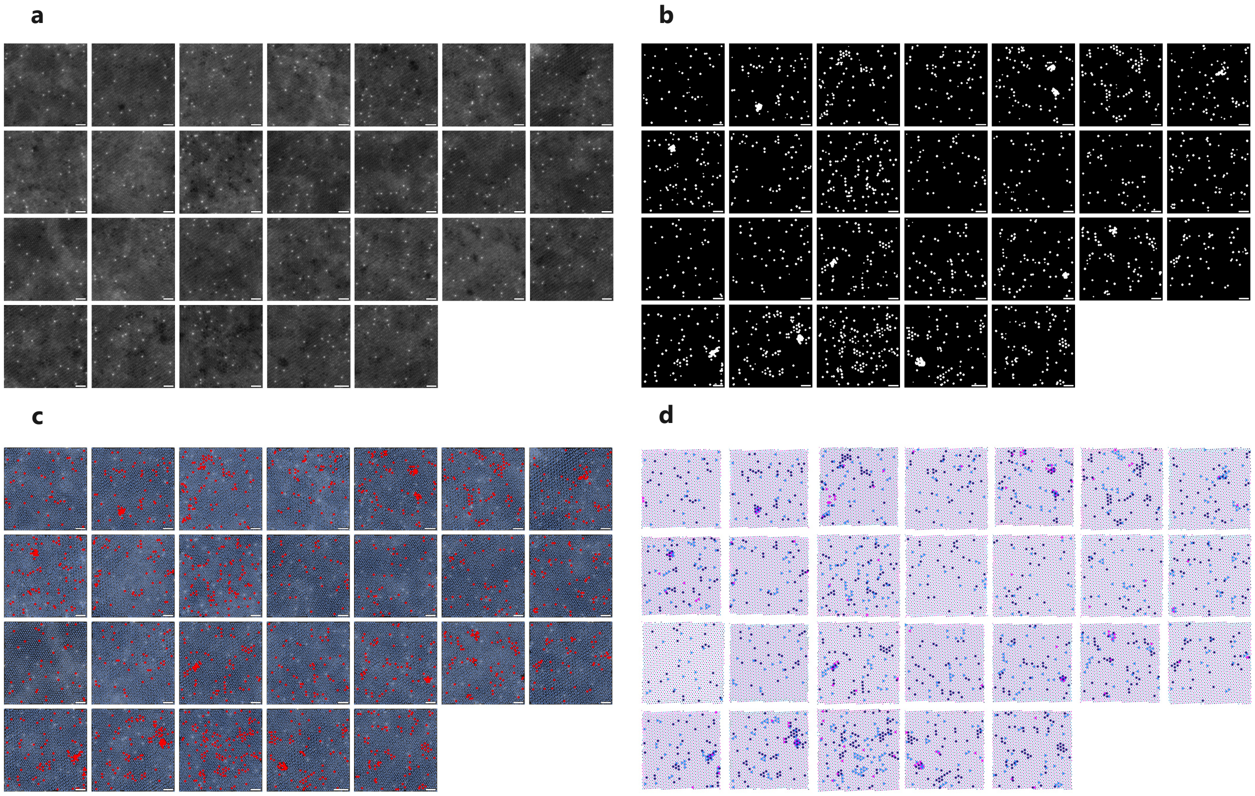

Supplementary Figure 12. **12.5% HF MXene images. a** STEM images of MXenes etched with 12.5% HF. **b** Neural network-outputted masks from the defect finder NN. White blobs indicate predicted defects. **c** All located atoms (blue) and defects (red). **d** Atoms and defects colored by layer; M'_{max} (dark blue), M'_{min} (light blue), M" (pink). Scale bars 1 nm.

Supplementary References

1. Nord, M., Vullum, P. E., MacLaren, I., Tybell, P. T. M. & Holmestad, R. Atomap: a new software tool for the automated analysis of atomic-resolution images using two-dimensional Gaussian fitting. *Adv. Struct. Chem. Imaging* **3**, 9 (2017).
2. Sang, X. *et al.* Atomic defects in monolayer titanium carbide ($Ti_3C_2T_x$) MXene. *ACS Nano* **10**, 9193–9200 (2016).
3. PlotDigitizer. PlotDigitizer (version 3.1.6). https://plotdigitizer.com (2026).
